# Supplementary material for: Qualitative and Quantitative Analyses of Sialyl O-Glycans in Milk-Derived Sialylglycopeptide Concentrate
Source: Foods. 2024 Sep 2;13(17):2792. doi: 10.3390/foods13172792 (PMC11395400; doi:10.3390/foods13172792)
Supplement: Supplementary file 1 [file foods-13-02792-s001.zip › foods-3169947-supplementary.pdf]

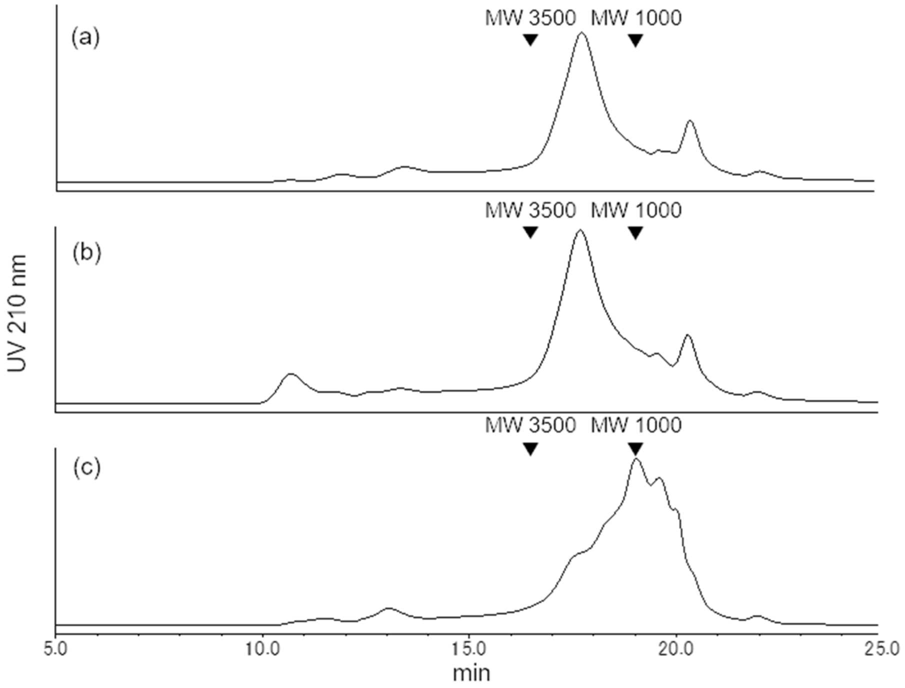

Figure S1. Size-exclusion chromatograms of the milk-derived sialylglycopeptide (MSGP) concentrate and MSGP concentrate treated with enzymes. MSGP concentrate (a), MSGP concentrate treated with proteinase K (b), and MSGP concentrate treated with proteinase K after treatment with neuraminidase and *O*-glycosidase (c) were applied to a tandem combination of Inertsil Diol and Inertsil WP300 Diol columns (GL Sciences) and eluted with 40% (vol/vol) acetonitrile containing 0.01% trifluoroacetic acid at a flow rate of 0.3 mL/min. Absorbance was monitored at 210 nm. The inverted triangle symbol indicates the retention time of the molecular weight (MW) calculated from β-lactoglobulin (MW: 18,277), *α*-lactalbumin (MW: 14,146), aprotinin (MW: 6512), sialylglycopeptide (from egg yolk, MW: 2866), and oxytocin (MW: 1007).

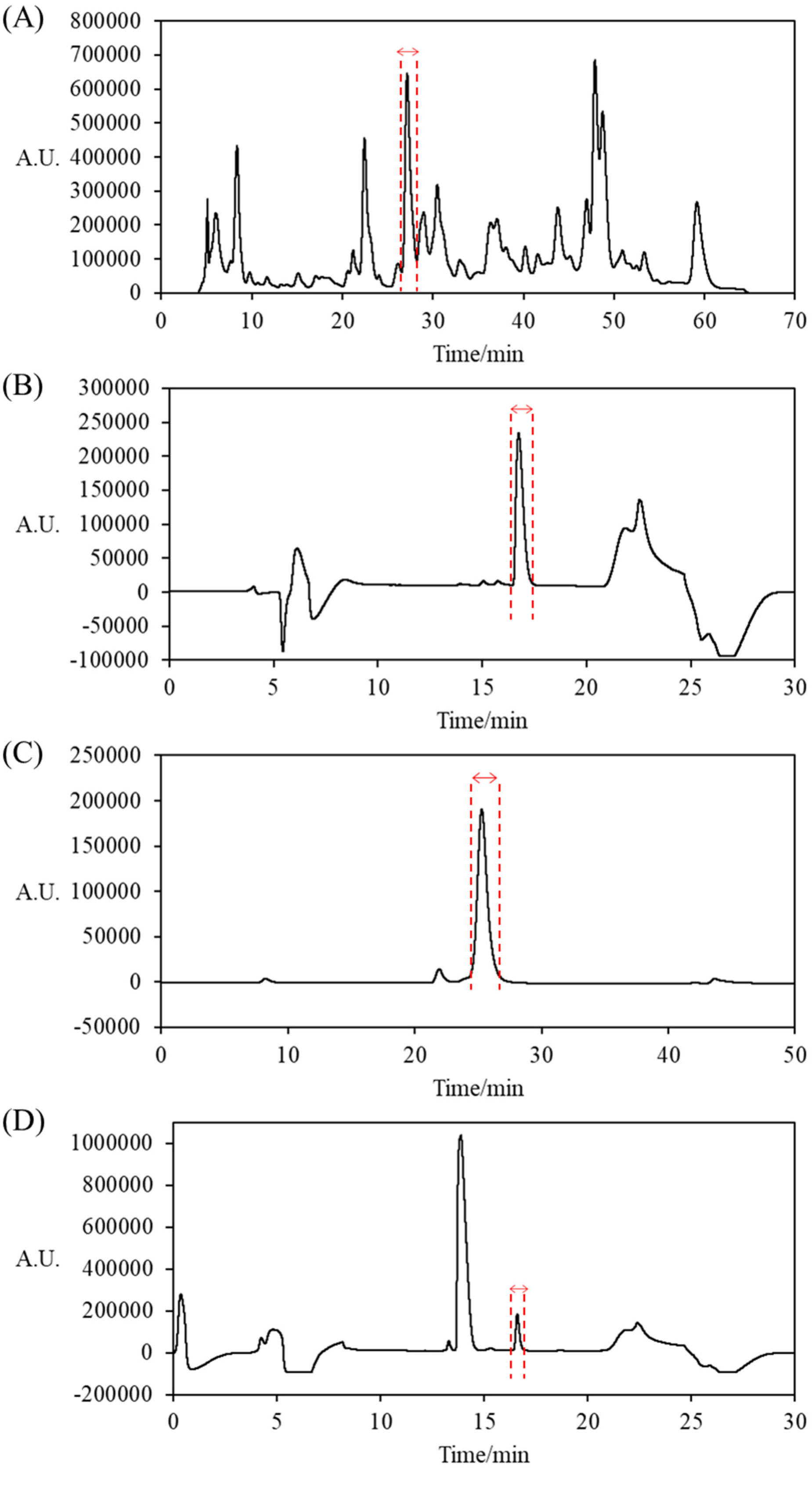
(A)
800000
700000
600000
500000
A.U. 400000
300000
200000
100000
0
0 10 20 30 40 50 60 70
Time/min
(B)
300000
250000
200000
150000
A.U. 100000
50000
0
-50000
-100000
0 5 10 15 20 25 30
Time/min
(C)
250000
200000
150000
A.U. 100000
50000
0
-50000
0 10 20 30 40 50
Time/min
(D)
1000000
800000
600000
A.U. 400000
200000
0
-200000
0 5 10 15 20 25 30
Time/min

Figure S2. HPLC chromatogram of the preparation of homogeneous *O*-glycopeptides with sialyl core 1 *O*-glycans. (A) HPLC chromatogram of the MSGP concentrate. The MSGP concentrate was loaded onto a HILIC amino column (NH2P-90 20F) and eluted using NaH_2PO4 solution. The peak containing 8-aa *O*-glycopeptides (GEPTSTPT) with a disialyl core 1 *O*-glycan (Neu5Acα2,3Galβ1,3(Neu5Acα2,6)GalNAc) at 27.2 min was fractionated as semi-purified disialyl *O*-glycopeptide. (B) HPLC chromatogram of semi-purified disialyl *O*-glycopeptides. Semi-purified disialyl *O*-glycopeptides were loaded onto the C18 RP column (YMC-Pack ODS-A) and eluted using H_2O/acetonitrile–0.1% formic acid. The peak containing 8-aa *O*-glycopeptides with a disialyl core 1 *O*-glycan at 16.7 min was fractionated. (C) HPLC chromatogram of 8-aa *O*-glycopeptides with a branched monosialyl *O*-glycan (Galβ1,3(Neu5Acα2,6)GalNAc). After treating the disialyl *O*-glycopeptides with α2,3-specific neuraminidase, the sample was loaded onto a HILIC amino column, and the peak containing the branched monosialyl core 1 *O*-glycan at 25.3 min was fractionated. (D) HPLC chromatogram of 8-aa *O*-glycopeptides with a linear monosialyl *O*-glycan (Neu5Acα2,6Galβ1,3GalNAc). After treating the disialyl *O*-glycopeptides with non-specific neuraminidase, the sample was loaded onto a C18 RP column, and the peak containing linear monosialyl core 1 *O*-glycopeptide at 16.6 min was fractionated. The peak at 14.1 min correspond to 8-aa *O*-glycopeptides with an asialyl *O*-glycan (Galβ1,3GalNAc). All chromatograms were monitored at a wavelength of 214 nm. Red arrows indicate the fractionated peaks in the chromatograms.

Table S1. Glycopeptides detected from the MSGP concentrate

No.	Protein	Peptide sequence*	Proposed glycan**
1	GMP	DKTEIPTIN	**3/4**
2		DKTEIPT	**5**
3		DKTEIPTIN	**5**
4		DKTEIPTINT	**5**
5		KTEIP*T*INT	**5**
6		TEIP*T*INT	**3/4**
7		TEIP*T*INT	**5**
8		EIP*T*IN	**3/4**
9		EIP*T*INT	**3/4**
10		EIP*T*IN	**5**
11		ASGEPTSTPT	**3/4**
12		ASGEPTSTPT	**3/4**
13		ASGEP*T*STPT	**5**
14		ASGEP*T*STPTIE	**5**
15		ASGEPTS*T*PTIE	**5**
16		ASGEPTSTPT	**6**
17		ASGEPTSTPT	**6**
18		ASGEPTSTPT	**6**
19		ASGEPTSTPT	**1 + 5**
20		ASGEPTSTPTIE	**3/4 + 5**
21		SGEPTSTPT	**3/4**
22		SGEPTSTPT	**3/4**
23		SGEPTSTPTIE	**3/4**
24		SGEPTSTPTIE	**3/4**
25		SGEPTSTPTIE	**3/4**
26		SGEP*T*STPT	**5**
27		SGEPTS*T*PTIE	**5**
28		SGEP*T*STPTIE	**5**
29		SGEPTSTPTTE	**5**
30		SGEPTSTPT	**6**
31		SGEPTSTPT	**1 + 5**
32		SGEPTSTPTIE	**3/4 + 5**

33	SGEPTSTPTIE	**3/4 + 5**
34	SGEPTSTPITE	**5 + 5**
35	GEP*T*STPT	**3**
36	GEP*T*STPT	**4**
37	GEPTSTPTIE	**3/4**
38	GEPTSTPTTE	**3/4**
39	GEPTSTPTTE	**3/4**
40	GEPTSTPT	**1 + 3/4**
41	GEPTS*T*PT	**5**
42	GEP*T*STPT	**5**
43	GEPTS*T*PTIE	**5**
44	GEP*T*STPTIE	**5**
45	GEPTS*T*PTTE	**5**
46	GEP*T*STPTTE	**5**
47	GEP*T*STPT	**6**
48	GEP*T*STPT	**6**
49	GEP*T*STPT	**6**
50	GEPTSTPTIE	**6**
51	GEP*T*STPTTE	**6**
52	GEPTSTPTIE	**2 + 5**
53	GEPTSTPTIE	**2 + 5**
54	GEPTSTPTIE	**2 + 5**
55	GEPTSTPTIE	**2 + 5**
56	GEPTSTPTIE	**2 + 5**
57	GEPTSTPTIE	**2 + 5**
58	GEPTSTPTIE	**2 + 5**
59	GEPTSTPTTE	**2 + 5**
60	GEPTSTPT	**3/4 + 5**
61	GEPTSTPTIE	**3/4 + 5**
62	GEPTSTPTIE	**3/4 + 5**
63	GEPTSTPTIE	**3/4 + 5**
64	GEPTSTPTIE	**3/4 + 5**
65	GEPTSTPTTE	**3/4 + 5**
66	GEPTSTPTTEA	**3/4 + 5**
67	GEPTSTPTIE	**5 + 5**

68	GEPTSTPTIE	**5 + 5**
69	GEPTSTPTTE	**5 + 5**
70	GEPTSTPTTEA	**5 + 5**
71	EPTSTPT	**3/4**
72	EPTSTPT	**3/4**
73	EP*T*STPT	**5**
74	EPTSTPTIE	**5**
75	EPTSTPTIE	**5**
76	STPTI	**5**
77	STPT	**5**
78	S*T*PTIE	**5**
79	S*T*PTTE	**5**
80	STPT*T*EA	**5**
81	TPT*T*EA	**5**
82	*T*PTTEAVE	**5**
83	TPTTEAVE	**6**
84	AVES*T*VAT	**3**
85	AVES*T*VAT	**4**
86	AVES*T*VA	**5**
87	AVES*T*VAT	**5**
88	AVES*T*VAT	**6**
89	AVE*ST*VAT	**1 + 5**
90	AVESTVAT	**2 + 5**
91	AVESTVAT	**3/4 + 5**
92	AVESTVAT	**5 + 5**
93	VESTVAT	**3/4**
94	VESTVAT	**3/4**
95	VES*T*VA	**5**
96	VES*T*VAT	**5**
97	VES*T*VAT	**6**
98	VES*T*VAT	**7**
99	VE*ST*VAT	**1 + 5**
100	VE*ST*VAT	**2 + 5**
101	VESTVAT	**3/4 + 5**
102	VE*ST*VAT	**5 + 5**

103		ESTVAT	**3/4**
104		ES*T*VA	**5**
105		ES*T*VAT	**5**
106		ES*T*VAT	**6**
107		ES*T*VAT	**7**
108		ES*T*VAT	**7**
109		ES*T*VAT	**7**
110		E*ST*VAT	**1 + 5**
111		ESTVAT	**2 + 5**
112		S*T*VAT	**3**
113		STVAT	**3/4**
114		S*T*VA	**5**
115		S*T*VAT	**5**
116		S*T*VA	**6**
117		S*T*VAT	**6**
118		STVAT	**2 + 3/4**
119		STVAT	**7**
120		STVAT	**7**
121		EASPE	**5**
122		ASPE	**3**
123		SPPEINTVQ	**5**
124		SPPEINTVQVT	**5**
125		INTVQVT	**5**
126		V*T*ST	**5**
127		V*T*STAV	**5**
128		TS*T*AV	**5**
129		*T*STAV	**5**
130		S*T*AV	**5**
131		S*T*AV	**6**
132	Osteopontin	DFPTDIPTIA	**5 + 5**
133		DFPTDIPTIA	**5 + 7 (6 + 6)**
134	Glycosylation-dependent cell adhesion molecule 1	AQP*T*DAS	**5**
135		AQP*T*DAS	**6**
136		TTEHTPS	**8**
137		TTEHTPSDA	**9**

138	(GlyCAM-1)	T*T*EHTPSDA	**8**
139		*N*ATL	**10**
140		*N*ATL	**11**
141		*N*ATL	**12**

*Glycosylation sites (serine or threonine) determined through electron transfer dissociation (ETD) analysis are shown in red.

** Proposed glycans **1–7** correspond to the glycan types in Figure 2. The structures of glycan **8–12** are as follows: **8**: Galβ1,3(Galβ1,4GlcNAcβ1,6)GalNAc; **9**: Neu5Acα2,3Galβ1,3(Galβ1,4GlcNAcβ1,6)GalNAc; **10**: Neu5Acα2,6Galβ1,4GlcNAcβ1,2Manα1,3(GlcNAcβ1,2Manα1,6)Manβ1,4GlcNAcβ1,4GlcNAc; **11**: Neu5Acα2,6Galβ1,4GlcNAcβ1,2Manα1,3(GlcNAcβ1,2Manα1,6)Manβ1,4GlcNAcβ1,4(Fucα1,6)GlcNAc; **12**: Neu5Acα2,6Galβ1,4GlcNAcβ1,2Manα1,3(Galβ1,4GlcNAcβ1,2Manα1,6)Manβ1,4GlcNAcβ1,4GlcNAc.
